# Establishment of Reference Measurement Procedure for *TP53* R175H/R248W Detection and a Novel Preparation Method for ctDNA Reference Material

**DOI:** 10.3390/genes16050576

**Published:** 2025-05-14

**Authors:** Yanru Tang, Chunyan Niu, Jiejie Zhang, Lianhua Dong, Jingya Yang

**Affiliations:** 1 College of Food Science and Technology, Shanghai Ocean University, Shanghai 201306, China; luckytang6@163.com (Y.T.); jjzhang@shou.edu.cn (J.Z.); 2 Center for Advanced Measurement Science, National Institute of Metrology, Beijing 100013, China; niuchy@nim.ac.cn (C.N.); lianhuadong@126.com (L.D.); 3 Marine Biomedical Science and Technology Innovation Platform of Lin-gang Special Area, Shanghai 201306, China

**Keywords:** ctDNA, dPCR, *TP53*, gene mutation

## Abstract

Background/Aims: Circulating tumor DNA (ctDNA) is becoming a valuable cancer biomarker for clinical decision-making. Nevertheless, the lack of quality control materials to assess the reliability of test results remains a challenge. This study aimed to establish digital PCR (dPCR) assays for detecting *TP53* variants (R175H and R248W) and develop a preparation method for ctDNA reference materials to improve detection reliability. Methods: Two dPCR assays targeting TP53-R175H and TP53-R248W variants were developed and validated for repeatability, sensitivity, and linearity. Additionally, a ctDNA reference material preparation protocol was developed by digesting nucleosomes from cultured cancer cell lines with micrococcal nuclease, followed by magnetic beads purification. The size distribution and quality of the generated ctDNA fragments was analyzed, and the developed dPCR assays were applied to detect the variants in the ctDNA samples. Results: The dPCR assays demonstrated high repeatability (RSD of 0.16% to 7.65%) and excellent linearity (R^2^ values of 1.0000 and 0.9981) across variant allele frequencies of 50%–0.1%. The limits of detection (LOD) and quantification (LOQ) were 0.143% (R175H) and 0.092% (R248W). The ctDNA reference materials exhibited single dominant peaks at 128 bp (R175H) and 143 bp (R248W). The dPCR assays successfully detected variants in these reference materials, confirming their applicability for ctDNA samples. onclusion: Firstly, accurate measurement procedures for TP53-R175H and TP53-R248W variants based on dPCR were established in this study. Furthermore, a protocol for preparing ctDNA reference material was established here. By digesting nucleosomal DNA derived from cancer cell lines with micrococcal nuclease, this method can closely mimic the properties of clinical ctDNA. The dPCR method and ctDNA reference material preparation approach established here could be used in ctDNA detection and for improving its reliability.

## 1. Introduction

Tumor biomarkers are essential for the effective management of cancer patients in areas such as diagnosis, recurrence monitoring, treatment target identification, drug resistance mechanisms, and prognosis evaluation [1,2,3]. The peripheral blood of individuals with cancer contains cellular products derived from both primary tumors and metastatic sites, including circulating tumor cells (CTCs) [4], nucleic acids [5,6,7], extracellular vesicles, and proteins [1]. These components can reflect genetic alterations in cancer cells and serve as valuable biomarkers for tumor detection.

Among these blood-derived biomarkers, circulating tumor DNA (ctDNA) has garnered significant attention in recent years, characterized by a length of 90–150 base pairs [8] and a half-life ranging from 15 min to 2 h [9]. It can provide real-time insights into the status of tumors within the body, and the detection of ctDNA can also unveil various genetic alterations, including single-nucleotide variations and copy number variations [10], as well as methylation modifications [11]. Consequently, it is increasingly being used to assist in clinical decision-making [12].

*TP53* is a tumor suppressor gene that encodes the tumor suppressor factor p53 protein [13,14]. Currently, studies have indicated that the *TP53* gene is associated with various tumors and influences cell proliferation and tumor growth by participating in processes such as DNA repair, senescence, cell cycle arrest, autophagy, and apoptosis [15]. In clinical practice, codons R175H and R248W serve as primary targets for *TP53* mutation screening due to their high prevalence across diverse malignancies. The hot spot mutation R175H site is found to be located near the zinc-binding site of the DNA binding interface, leading to protein structure alterations and promoting tumor cell invasion and migration [16,17,18]. On the other hand, R248W occurs in amino acids that interact with DNA, resulting in the inability of p53 to bind to DNA and the loss of tumor suppressive function or the acquisition of functions that facilitate tumor development [19,20]. *TP53* functional deficiencies not only lead to tumor development but also compromise the response of malignant cells to anti-cancer drugs, making the *TP53* gene a promising therapeutic target [21].

ctDNA requires quantitative detection methods with a high sensitivity and accuracy. Next-generation sequencing (NGS) enables the comprehensive profiling of genomic variants, while there are still risks to its reliability due to the variability in amplification efficiency or biases in bioinformatics algorithms. Reference materials are needed for assessing the analytical performance of various NGS assays. Digital PCR (dPCR), a widely used method in nucleic acid quantification, overcomes certain limitations through partitioned amplification in microreaction units and single-molecule counting, providing absolute quantification without the need for standard curves. dPCR attains a detection sensitivity below 0.01%, making it particularly suitable for low-abundance ctDNA detection. Moreover, its robust tolerance to inhibitors in complex matrices (e.g., blood and urine) ensures reliable performance in suboptimal-quality specimens. This study developed dPCR assays for the quantification of two clinically relevant mutations (R175H and R248W) in the *TP53* tumor suppressor gene. These dPCR assays were subsequently utilized in the development of reference materials.

Current mainstream external quality control programs, such as UKNEQAS [22] (United Kingdom National External Quality Assessment Service) and IQN Path [23] (International Quality Network for Pathology), have established standardized quality control criteria for clinical ctDNA detection. However, the detection process still requires available reference materials for internal quality control [23]. Current ctDNA reference materials predominantly utilize mechanical methods (e.g., ultrasonication) or PCR-based synthetic approaches. However, ultrasonication-induced high-energy shearing generates nonspecific terminal damage, while PCR-based methods may introduce biases, potentially have sequence errors, lack epigenetic information, and fail to fully capture heterogeneity.

To address these needs, this study applied genomic DNA from cell lines and developed an enzymatic reaction system to generate ctDNA reference materials mimicking clinical specimens. Micrococcal nuclease directly cleaved nucleosomal DNA from cancer cell lines, followed by magnetic-bead-based purification to yield fragments size-matched to clinical ctDNA. In contrast to conventional methods, our enzymatic approach utilizes human genomic DNA as the substrate, thereby retaining the intrinsic native epigenetic features inherent to clinical ctDNA.

## 2. Materials and Methods

### 2.1. Cell Lines

The TP53-R175H-positive cell line (SK-BR-3) and TP53-R248W-positive cell line (MIA PaCa-2) were purchased from Zhejiang Meisen Cell Technology Co., Ltd. (Hangzhou, Zhejiang Province, China). Both cell lines were cultured in DMEM medium in a 5% CO_2_ incubator at 37 °C. Medium for SK-BR-3 cell line contained 15% fetal bovine serum (FBS, Meisen cell, cat# 00207150) and 1% penicillin streptomycin solution (PS, Meisen cell, cat# 002072), while medium for MIA PaCa-2 cell line contained 10% fetal bovine serum and 1% penicillin streptomycin solution. When cells reached 80–90% of the bottom of T-175 culture flask, culture medium were removed and phosphate-buffered saline (PBS) were added to wash the cells. Cells were detached from the flask surface using 0.25% Trypsin-EDTA (1×) (Meisen cell, cat# 002006) and complete culture medium were added to terminate digestion. Cell pellets were collected by centrifugation at 1500× *g*/min for 5 min and stored at −80 °C for further use.

Variants in cell lines were confirmed by Sanger sequencing of PCR products containing targeted mutation. Genomic DNA was exacted from cells using genomic DNA extraction kit (TIANGEN, Beijing, China) and used for PCR reaction. PCR amplification was carried out in a total 50 µL reaction volume using TransStart Fsat Pfu DNA Polymerse kit (TransGen Biotech, Beijing, China) on VeritiPro 96-Well Thermal Cycler (Thermo Fisher Scientific, Waltham, MA, USA) for 35 cycles according to the conditions specified by the manufacturer. Primers used for PCR amplification for Sanger sequencing are listed in Table S1. PCR products were used for Sanger sequencing by BGI (Shenzhen, Guangdong Province, China). The Sanger sequencing results are shown in Table S2 and Figure S1.

### 2.2. Digital PCR Assays

Primers and probes used in this study for TP53-R175H and TP53-R248W mutation were synthesized and purified by Sangon Biotech (Shanghai, China). The primer and probe sequences are shown in Table 1.

All the dPCR reactions were performed using Bio-Rad QX200 digital PCR system, which are based on optimization of annealing temperature and primer/probe concentration (Supplementary Materials and Figures S2–S5). For dPCR reaction, 20 μL of mixtures were prepared, containing 2 μL of template DNA, 10 μL of 2 × ddPCR supermix for probes (Bio-Rad Laboratories, Hercules, CA, USA), 10 μL of primers (final concentration 500 nM), 1 μL of wild-type (WT) probe (final concentration 250 nM), 1 μL of mutation (MU) probe (final concentration 250 nM), and 5 μL of water. Droplets were generated on QX200 droplet generator (Bio-Rad Laboratories, Hercules, CA, USA). PCR was performed at 95 °C for 10 min; 94 °C for 30 s, and 56 °C for 1 min with 40 cycles; and 98 °C for 10 min using thermocycler (VeritiPro, Thermo Fisher). Thermal-cycled plate was analyzed on QX200 droplet reader (Bio-Rad Laboratories, Hercules, CA, USA). Data analysis was performed with the QuantaSoft software (version 1.7.4., Bio-Rad).

### 2.3. Assessment of Dynamic Range and Repeatability

To assess the dynamic range and repeatability of the established assays, wild-type and mutant plasmids were mixed using a weight-based approach to achieve variant allele frequencies (VAFs) of 50%, 20%, 5%, 1%, 0.2%, and 0.1%. These samples were then subjected to dPCR measurement with 3–6 replicates for each VAF. A linear relationship between measured results of dPCR and gravimetric results was established. Repeatability of the assays was determined as the relative standard deviation (RSD) of VAF.

### 2.4. Determination of Limit of Blank (LOB), Limit of Detection (LOD), and Limit of Quantification (LOQ)

Determination of the method’s LOB was performed according to IFCC’s guideline EP17-A [24] by measuring 30 replicates of wild-type plasmid and 30 of wild-type genomic DNA samples. Limit of detection (LoD) is the lowest amount of analyte in a sample, and limit of quantitation (LOQ) is the lowest amount of analyte in a sample that can be quantitatively determined with stated acceptable precision and trueness, under stated experimental conditions [24]. A series of samples with low-level VAF were prepared by mixing the mutant and wild-type plasmid at various ratios through gravimetric method, achieving VAF of 0.2%, 0.1%, 0.05%, 0.02%, and 0.01%. Each level of samples was measured by dPCR with 12 replicates to determine the LOD and LOQ of the dPCR method. The detection of a replicate was considered positive when the measured VAF was higher than its corresponding LOB. LOD_95%_ were calculated by Probit regression analysis with a 95% repeatable probability using MedCalc v23.09 (MedCalc Software Ltd., Ostend, Belgium).

### 2.5. Preparation of ctDNA Reference Materials

Fragmented DNA reference materials were prepared by digesting nucleosomes using Micrococcal Nuclease (MNase) (New England Biolabs, Ipswich, MA, USA, cat# M0247S) to mimic ctDNA samples. The nuclear fraction was first extracted from cultured cell lines. Then, 1 mL of PBS were added to the collected cell pellets to wash them twice by centrifugation at 4 °C and 800× *g* for 10 min. Then, 1 mL of pre-cooled NP-40 was added, and cells resuspended, and incubated on ice for 10 min to obtain cell lysis. Nuclear fraction was extracted using a cell nucleus extraction kit (Beijing Box Biotech Co., Ltd., Beijing, China, cat# AKNA002-1) according to the protocol of manufacture.

The extracted cell nuclear fraction was subjected to enzymatic digestion using MNase. The digestion system consisted of 100 μL of ddH_2_O, 10 μL of 10 × MNase Buffer, 10 μL of 10 × protease inhibitors, 0.5 μL of 20 mg/mL recombinant albumin, and 0.5 μL of MNase (2 × 10^6^ gel units/mL). The mixture was incubated at 37 °C for 10 min for the digestion reaction and kept at 65 °C for 10 min to inactivate the enzymes. Following this step, the digested cell nucleus was diluted with 2-fold volume of water before adding in a solution containing 3 μL of proteinase K solution (20 g/L) and 10 μL of 20% SDS solution. This preparation was then incubated at 60 °C for 20 min.

### 2.6. Purification of ctDNA Using Magnetic Beads

**Figure 1:**
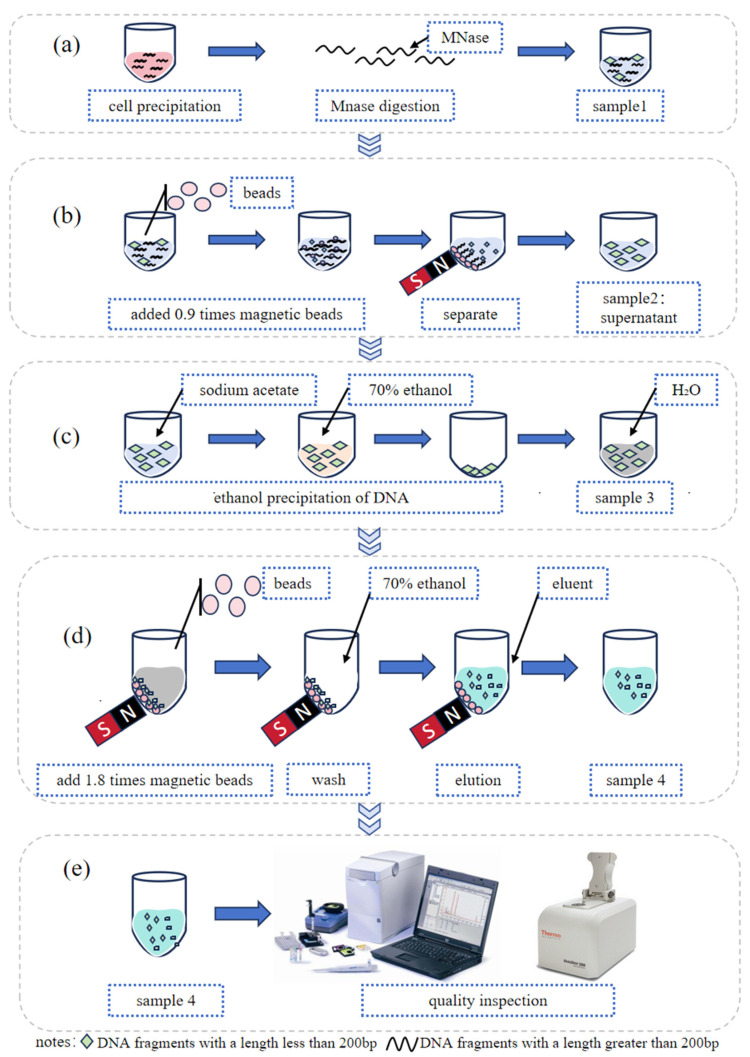
Flow chart of ctDNA reference material preparation: (**a**) extracting cell nuclei from cell sediment; (**b**) removing DNA fragments longer than 200 bp; (**c**) ethanol precipitation of DNA for removing impurities from the system; (**d**) extracting the target fragment; and (**e**) quality inspection on the extracted target fragment.

The digested DNA fragments were further purified using AMPure™ XP Beads (Beckman Coulter, Brea, CA, USA, cat# A63881) according to the protocol of manufacture. Two rounds of purification were performed to obtain a single peak of DNA fragments. In the first round, 0.9 times the bead volume was used, followed by 1.8 times the volume in the second round. The size of was detected using Qsep portable capillary electrophoresis instrument (Hangzhou Houze Biotechnology Co., Ltd., Hangzhou, Zhejiang Province, China) or Agilent 2100 (Agilent Technologies Co., Ltd., Beijing, China). The schematic representation of the ctDNA reference material preparation process is presented in Figure 1.

## 3. Results and Discussion

### 3.1. Validation of Duplex dPCR Assays for TP53 R175H and R248W

In the detection of the TP53-R175H and TP53-R248W mutation sites using the dPCR method, the single-nucleotide variation characteristic of these point mutations results in only a single-base difference between the wild-type and mutant probe sequences. This minimal distinction renders them prone to cross-interference during PCR amplification. To validate the accuracy of the experimental design, it is essential that we confirm the specificity of the probes during the method establishment.

In this study, duplex probes (WT + MU) targeting both mutant and wild-type sequences were employed to analyze four sample types: (1) the mutant plasmid harboring the target mutations, (2) the wild-type plasmid lacking the mutations, (3) mixed samples containing both mutant and wild-type alleles, and (4) nucleic-acid-free ddH_2_O controls. Probe specificity was rigorously verified based on PCR amplification outcomes.

**Figure 2:**
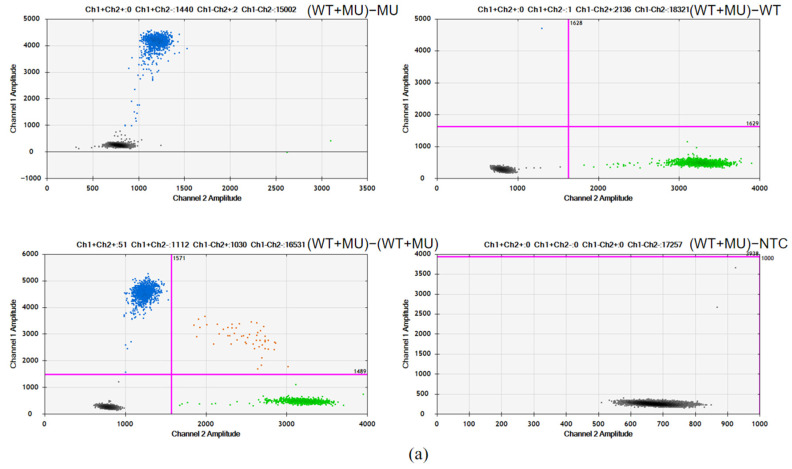

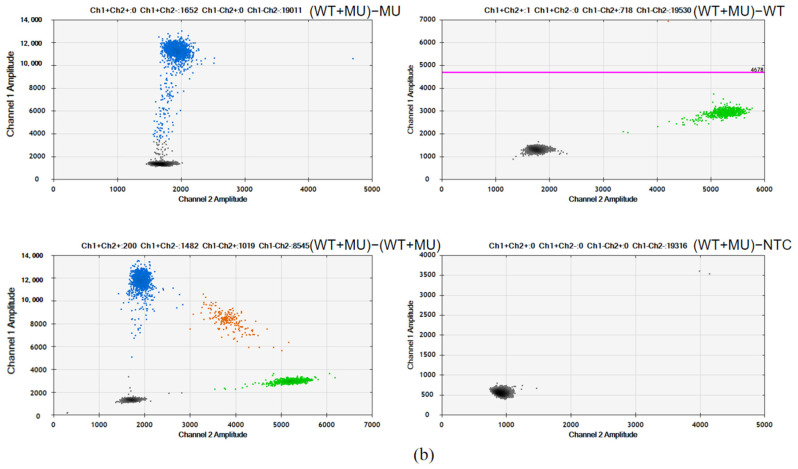
Specificity assessment of TP53-R175H (**a**) and R248W (**b**): (WT + MU) − MU, duplex probes with mutation plasmid; (WT + MU) − WT, duplex probes with wild-type plasmid; (WT + MU) − (WT + MU), duplex probes with wild-type and mutation plasmids; (WT + MU) − NTC, duplex probes with no templates.

As shown in Figure 2, for both R175H and R248W, the amplification of the mutant plasmid with duplex probes only resulted in a fluorescence signal in the FAM channel (MU probe), while no signal was observed in the VIC channel (WT probe), indicating the specific binding and amplification of the mutant sequences by the MU probe. Conversely, the amplification of the wild-type plasmid with duplex probes only yielded a fluorescence signal in the VIC channel, demonstrating the specific binding and amplification of the wild plasmid by the WT probe. Furthermore, the simultaneous amplification of mutant and wild-type plasmids with duplex probes produced distinct fluorescence signals in both FAM and VIC channels, clearly separated into four clusters, confirming the absence of cross-reactions between them and demonstrating the good specificity of the dPCR assays.

### 3.2. Dynamic Range and Repeatability of the Assays

**Figure 3:**
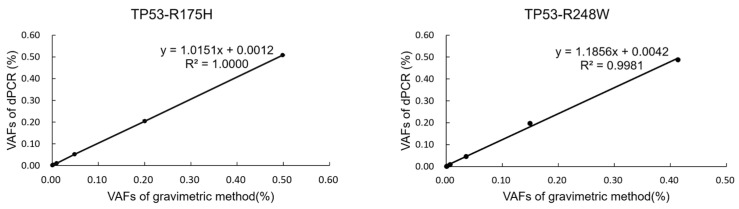
Dynamic range of TP53-R175H and TP53-R248W.

The dPCR results of six mixed samples with a series of VAFs, prepared using the gravimetric method, demonstrated a strong linear correlation between the dPCR measurement and gravimetric result, with R^2^ values of 1.0000 and 0.9981 for TP53-R175H and TP53-R248W, respectively (Figure 3). These findings suggest that the established dPCR methods exhibit a high accuracy in detecting the VAF of TP53-R175H and TP53-R248W across a range from 0.1% to 50%.

The repeatability of the quantitative PCR reaction reflects the stability and reliability of the established dPCR method. As such, the RSD was determined for the six mixed samples with varying VAFs, as shown in Table 2. The RSD values ranged from 0.23% to 7.65% for TP53-R175H and from 0.16% to 3.69% for TP53-R248W when the average mutation abundance fell within the range of 0.10–50.00%. These results demonstrate that the established dPCR detection methods for TP53-R175H and TP53-R248W exhibit excellent repeatability.

### 3.3. Blank Limit, Detection Limit, and Quantification Limit

The wild-type plasmid and gDNA results were pooled together to determine the LOB. As the distribution of the measurement results from the 60 blank samples was non-Gaussian, the LoB was estimated using a non-parametric method. The LoB is the value of position (60 × (95/100 + 0.5)) = 57.5, which is (57th result + 58th result)/2, so the LOB of TP53-R175H and TP53-R248W were determined to be 0.10% and 0.05% (Supplemental Tables S3 and S4).

**Figure 4:**
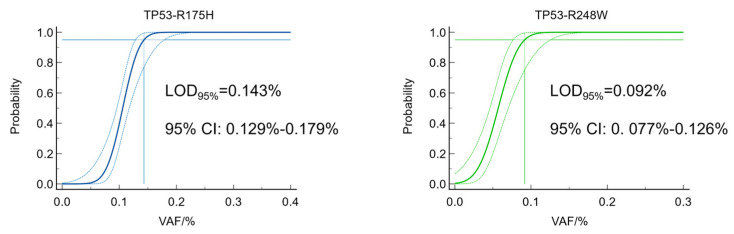
Probit regression analysis for determining the sensitivity of dPCR assays. Solid curves show the probability (range, 0 to 1) of a positive detection and the corresponding VAF, and dashed curves represent the 95% CI for the VAF. Limits of detection with 95% confidence (LOD_95%_) and 95% CIs were determined by a cross line at the 95% probability of positive detection.

Five levels of low VAF samples were measured to determine the LOD and LOQ. A replicate was considered positive when the measured VAF exceeded its corresponding LOB. Thus, the LOD_95%_ calculated from the Probit regression in VAFs was 0.143% for TP53-R175H and 0.092% for TP53-R248W (Figure 4 and Supplemental Table S5). The LOQ was determined by calculating the statistic Total Error = bias + 2SDs (SD is the sample standard deviation, and bias is the deviation between the measured value and the true value). If the Total Error is less than the preset target value, then LOQ = LOD. Thus, the LOQ of TP53-R175H and TP53-R248W were 0.143% and 0.092%, respectively.

### 3.4. Development of the Preparation of ctDNA Reference Materials

**Figure 5:**
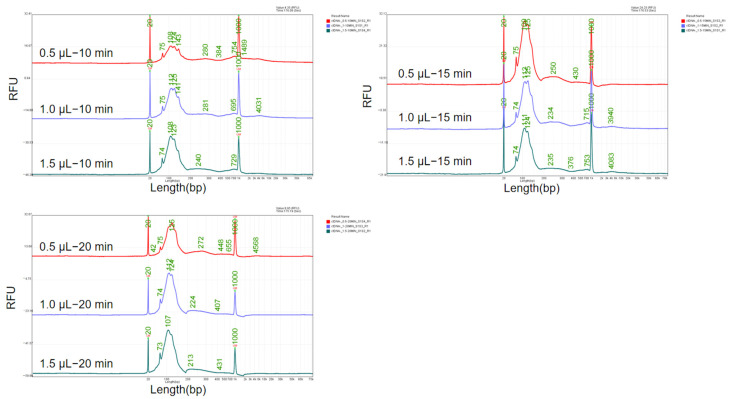
Optimization of enzyme concentration and digestion time of MNase. Various enzyme concentration (0.5 μL, 1 μL, and 1.5 μL) and digestion time (10 min, 15 min and 20 min) were tested.

MNase cleaves the linker arms between histones, resulting in a distinct 147 bp DNA band according a previous report [25]. It was utilized in this study for the preparation of fragmented DNA reference material mimicking ctDNA. During the research of the digestion process, we found that both the enzyme concentration and digestion time have an impact on the length of the obtained DNA fragments. Therefore, an orthogonal experiment was conducted using enzyme concentrations of 0.5 μL, 1.0 μL, and 1.5 μL of 2 × 10^6^ gel units/mL and digestion times of 10 min, 15 min, and 20 min. The sizes of the DNA fragments are shown in Figure 5.

By maintaining a constant enzyme digestion time and increasing the enzyme dosage, the fragment length decreases as the enzyme dosage increases. Similarly, when keeping the enzyme dosage constant and extending the enzyme digestion time, the fragment length also decreases with the extension of digestion time. Specifically, at an enzyme dosage of 0.5 μL and a digestion time of 10 min, the main peak length is from 108 bp to 143 bp, which aligns with the ctDNA size distribution. Therefore, the enzyme dosage of 0.5 μL of 2 × 10^6^ gel units/mL and the digestion time of 10 min were determined to be used for subsequent experiments.

**Figure 6:**
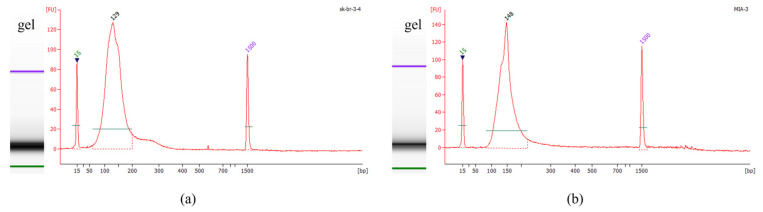
Fragmented DNA containing R175H (**a**) and R248W (**b**) mutation.

The optimized system for preparing ctDNA reference material was used to treat cell lines (the SK-BR-3 and MIA PaCa-2 cell lines) containing TP53-R175H and TP53-R248W, and the results are shown in Figure 6 and Table 3. Firstly, the concentration and purity of the ctDNA reference material were initially assessed using a Nanodrop 2000 UV–Vis spectrophotometer (Thermo Fisher Scientific, Waltham, MA, USA). As shown in Table 3, the A260/A280 ratios ranged between 1.8 and 2.0, while all A260/A230 ratios exceeded 2.0. These spectrophotometric measurements confirmed the absence of protein contaminants, enzymatic residues, salt ions, or other impurities in the prepared samples. Subsequently, the fragment length distribution and molecular purity of the ctDNA reference material were evaluated through Agilent 2100 Bioanalyzer chip electrophoresis. The electrophoretic profile presented in Figure 6 exhibited a single distinct peak without detectable fragments of alternative lengths, demonstrating a high molecular homogeneity that aligns with the essential characteristics of certified reference materials. The purified DNA fragments were 129 bp in length for R175H and 148 bp for R248W, with a single main peak, indicating that the system established for preparing the ctDNA reference material can be applicable for different cell lines.

### 3.5. Application of dPCR Assays in ctDNA Detection

**Figure 7:**
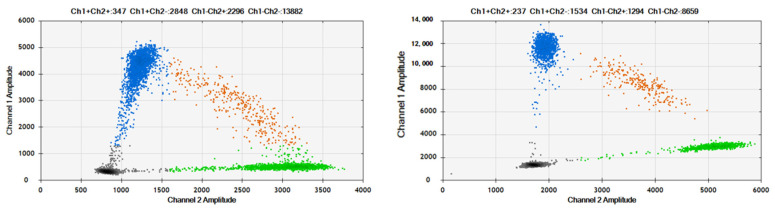
2D Plots of TP53-R175H and TP53-R248W assays in detection of ctDNA samples. The blue cluster (upper left quadrant) represents FAM-positive droplets, indicating successful amplification of TP53 R175H or R248W mutant alleles within these droplets, which emitted FAM fluorescence signals; The orange cluster (upper right quadrant) corresponds to double-positive droplets (FAM+/VIC+), where both mutant (TP53 R175H/R248W) and wild-type alleles were co-amplified, generating overlapping fluorescence signals from FAM and VIC channels; The gray cluster (lower left quadrant) denotes double-negative droplets, containing no detectable target molecules, as confirmed by the absence of fluorescence in both FAM and VIC channels; The green cluster (lower right quadrant) signifies VIC-positive droplets, reflecting amplification of wild-type *TP53* alleles exclusively, with fluorescence emission specific to the VIC channel.

The established dPCR assays for TP53-R175H and TP53-R248W were applied to detect mutations in the ctDNA samples. Two samples of ctDNA with VAFs of 50% for TP53-R175H and TP53-R248W were prepared by mixing ctDNA generated from wild-type (plcl6 cell line) and mutation-containing cell lines. The results are shown in Figure 7. Compared to plasmid DNA, the 2D plots of ctDNA amplification exhibit slight “rain” due to the fraction of DNA. However, they can still be distinguished by four clusters. The measured VAFs of the ctDNA were 50% for TP53-R175H and TP53-R248W, with an RSD of 0.89% and 1.84% (n = 6), respectively, which were consistent with the expected values.

## 4. Discussion

The ideal reference material for ctDNA should be directly isolated from patient-derived samples. However, in clinical settings, ctDNA present in plasma specimens from cancer patients is substantially diluted by an overwhelming abundance of non-neoplastic cell-free DNA. Moreover, ctDNA concentrations are markedly reduced in patients with a low tumor burden and specific cancer types, particularly gliomas and renal cell carcinomas. Consequently, the limited quantity of ctDNA in clinical samples precludes its utility as a sustainable source for reference materials. Thus, in this study, we developed the approach for preparing reference materials using fragmented DNA as a mimic of clinical ctDNA.

Existing ctDNA reference materials predominantly rely on synthetic oligonucleotides (e.g., gBlocks) or commercial controls, which suffer from limited scalability and high costs. Synthetic approaches lack physiological fragment heterogeneity, while ultrasonication-based methods require specialized equipment for controlled DNA shearing. In contrast, our micrococcal nuclease digestion method eliminates costly synthesis and specialized equipment dependency. This enzymatic strategy generates ctDNA fragments with size profiles closely resembling endogenous ctDNA and operates efficiently with standard laboratory instruments (e.g., centrifuges and thermal cyclers), enabling broad adoption in resource-limited settings. The enzymatic digestion method developed in this study can be extended to cell lines harboring additional mutations (e.g., EGFR or KRAS), thereby enabling the production of ctDNA reference materials containing specific mutation loci such as EGFR L858R or KRAS G12D.

This method has several inherent limitations. MNase and plasma nucleases (e.g., DNase I) generate distinct fragment end motifs. MNase preferentially cleaves nucleosome linker regions with an A/T sequence bias, producing fragments exhibiting a pronounced AT skew (GC content < 40%) that impair hybridization efficiency in GC-rich regions. In contrast, plasma nucleases yield stochastic cleavage patterns with a balanced terminal GC content, thereby better recapitulating the endogenous ctDNA fragmentation profiles. Consequently, MNase-digested genomic DNA may introduce systematic biases in hybridization-capture-based NGS assays.

While the developed dPCR assays and ctDNA reference material preparation methods demonstrate robust in vitro performance, their susceptibility to biological matrix interference (e.g., plasma protein binding or competitive inhibition by endogenous cfDNA) remains uncharacterized, and their clinical utility awaits empirical verification, and systematic biases introduced by alternative quantification methodologies (e.g., NGS) remain unverified. Future efforts will focus on the following: (1) spiking the ctDNA into biomimetic environments to validate its susceptibility; (2) applying the dPCR assays to ctDNA from patient samples; and (3) applying the ctDNA reference materials in multiplex detection systems, such as hybrid capture NGS.

## 5. Conclusions

In this study, dPCR assays for the quantification of two clinical variants of tumor suppressor gene *TP53* (R175H and R248W) were established. Firstly, the optimal annealing temperature (56 °C) and primer/probe concentration (250 nM/250 nM) were determined; then, the repeatability, linear range, and sensitivity of the detection methods were validated. The results indicated that these assay methods exhibited good repeatability (RSD ranging from 0.16% to 7.65%) and ideal linearity (R^2^ values of 1.0000 and 0.9981). The detection and quantification limits of R175H and R248W were 0.143% and 0.092%, respectively.

We prepared the quality control material for synthetic ctDNA with somatic mutations based on the use of MNase and magnetic beads. The optimal enzyme dosage of 0.5 μL of 2 × 10^6^ gel units/mL and the digestion time of 10 min were determined to be used for subsequent experiments. The system was verified to be applicable in both SK-BR-3 and MIA PaCa-2 cell lines, demonstrating the universality of the system.

The obtained ctDNA fragments were characterized by chip electrophoresis and measured by the established dPCR assays. Therefore, it has been demonstrated that our developed system is an ideal preparation protocol for artificially simulating ctDNA, which can be utilized to prepare high-quality ctDNA quality control and reference materials containing common mutations.

## Figures and Tables

**Table 1:** Primer and probe sequences of dPCR.

Variants	Type	Sequence (5′-3′)	Length of Amplicons
TP53-R175H	Forward	ACAGCACATGACGGAGGT	126 bp
	Reverse	GGAATCAGAGGCCTGGGG	
	MU-Probe	5′6-FAM-AGGCACTGCCCCCAC-3′MGB	
	WT-Probe	5′VIC-AGGCGCTGCCCCCAC-3′MGB	
TP53-R248W	Forward	AACAGTTCCTGCATGGGC	84 bp
	Reverse	GCAAGTGGCTCCTGACC	
	MU-Probe	5′6-FAM-CATGAACTGGAGGCC-3′MGB	
	WT-Probe	5′VIC-CATGAACCGGAGGCC-3′MGB	

**Table 2:** Repeatability of dPCR assays.

Dilutions	TP53-R175H		TP53-R248W	
	VAF/%	RSD/%	VAF/%	RSD/%
S1	50.76	0.23	48.83	0.72
S2	20.39	2.37	19.76	2.53
S3	5.22	1.52	4.66	0.16
S4	0.98	3.73	0.87	1.69
S5	0.25	2.23	0.23	2.25
S6	0.10	7.65	0.11	3.69

VAF, variant allele frequency. RSD, relative standard deviation.

**Table 3:** Quality control of ctDNA detected by Nanodrop 2000.

Variants	Cell Line	Cell Input Quantity (Unit)	Concentration (ng/μL)	ctDNA Production (μg)	A260/A280	A260/A 230
TP53-R175H	SK-BR-3	10^6^	322.50	1.45	1.91	2.23
TP53-R248W	MIA-PaCa-2	10^6^	143.70	1.29	1.88	2.44

## Data Availability

The original contributions presented in this study are included in the article/Supplementary Material. Further inquiries can be directed to the corresponding author(s).

## Supplementary Materials

The following supporting information can be downloaded at: https://www.mdpi.com/article/10.3390/genes16050576/s1, Figure S1: The Sanger sequencing results of cells; Figure S2: TP53-R175H annealing temperature optimization; Figure S3: TP53-R248W annealing temperature optimization; Figure S4: Optimization of primer and probe concentrations for TP53-R175H; Figure S5: Optimization of primer and probe concentrations for TP53-R248W; Table S1: Primers used for PCR amplification for Sanger sequencing; Table S2: Sanger sequencing amplification sequence; Table S3: Raw dPCR data for the LOB of the TP53-R175H; Table S4: Raw dPCR data for the LOB of the TP53-R248W; Table S5: Sensitivity analysis of the dPCR assays.
