# Supplementary material for: Establishment of Reference Measurement Procedure for TP53 R175H/R248W Detection and a Novel Preparation Method for ctDNA Reference Material
Source: Genes (Basel). 2025 May 14;16(5):576. doi: 10.3390/genes16050576 (PMC12110974; doi:10.3390/genes16050576)
Supplement: Supplementary file 1 [file genes-16-00576-s001.zip › genes-3636282-SM-updated.pdf]

Establishment of Reference Measurement Procedure for TP53 R175H/R248W Detection and a Novel Preparation Method for ctDNA Reference Material

Yanru Tang[1,2,†], Chunyan Niu[2,†], Jiejie Zhang[1,2], Lianhua Dong[2] and Jingya Yang[1,3,*]

[1] College of Food Science and Technology, Shanghai Ocean University, Shanghai 201306, China;

[2] Center for Advanced Measurement Science, National Institute of Metrology, Beijing 100013, China;

[3] Marine Biomedical Science and Technology Innovation Platform of Lin-gang Special Area, Shanghai 201306, China

*Correspondence: jyyang@shou.edu.cn

†These authors contributed equally to this work.

Table S1. Primers used for PCR amplification for Sanger sequencing.

Variants	Forward	Reverse
TP53-R175H	GCCAACTCTCTCTAGCTCGC	CCCCTACTGCTCACCTGGAG
TP53-R248W	TGCCACAGGTCTCCCCAAG	CCTGCTTGCTTACCTCGCTT

Table S2. Sanger sequencing amplification sequence.

Variants	Amplification sequence	Amplicon /bp
TP53-R175H	CGGGGGGGGGAGGTGCTTAACATGTTTGTTTCTTTGCTGCCG<br>TCTTCCAGTTGCTTTATCTGTTCACTTGTGCCCTGACTTTCAAC<br>TCTGTCTCCTTCCTCTTCCTACAGTACTCCCCTGCCCTCAACA<br>AGATGTTTTGCCAACTGGCCAAGACCTGCCCTGTGCAGCTGT<br>GGGTTGATTCCACACCCCCGCCCGGCACCCGCGTCCGCGCCA<br>TGGCCATCTACAAGCAGTCACAGCACATGACGGAGGTTGTGA<br>GGCACTGCCCCCACCATGAGCGCTGCTCAGATAGCGATGGTG<br>AGCAGCTGGGGCTGGAGAGACGACAGGGCTGGTTGCCCAGG<br>GTCCCCAGGCCTCTGATTCCTCACTGATTGCTCTTAGGTCTGG<br>CCCCTCCTCAGCATCTTATCCGAGTGGAAGGAAATTTGCGTG<br>TGGAGTATTTGGATGACAGAAACACTTTTCGACATAGTGTGG<br>TGGTGCCCTATGAGCCGCCTGAGGTCTGGTTTGCAACTGGGG<br>TCTCTGGGAGGAGGGGTTAAGGGTGGTTGTCAGTGGCCCTCC<br>AGGTG	590
TP53-R248W	GCGGGGGGGCAGCTCGTGGTGAGGCTCCCCTTTCTTGCGGAG<br>ATTCTCTTCCTCTGTGCGCCGGTCTCTCCCAGGACAGGCACAA<br>ACACGCACCTCAAAGCTGTTCCGTCCCAGTAGATTACCACTA<br>CTCAGGATAGGAAAAGAGAAGCAAGAGGCAGTAAGGAAATC<br>AGGTCCTACCTGTCCCATTTAAAAAACCAGGCTCCATCTACT<br>CCCAACCACCCTTGTCCTTTCTGGAGCCTAAGCTCCAGCTCCA<br>GGTAGGTGGAGGAGAAGCCACAGGTTAAGAGGTCCCAAAGC<br>CAGAGAAAAGAAAACTGAGTGGGAGCAGTAAGGAGATTCCC<br>CGCCGGGGATGTGATGAGAGGTGGATGGGTAGTAGTATGGA<br>AGAAATCGGTAAGAGGTGGGCCCAGGGGTCAGAGGCAAGCA<br>GAGGCTGGGGCACAGCAGGCCAGTGTGCAGGGTGGCAAGTG<br>GCTCCTGACCTGGAGTCTTCCAGTGTGATGATGGTGAGGATG<br>GGCCTCCAGTTCATGCCGCCCATGCAGGAACTGTTACACATG<br>TAGTTGTAGTGGATGGTGGTACAGTCAGAGCCAACCTAGGAG<br>ATAACACAGGCCCAAGATGAGGCCAGTGCGCCTTGGGGGCC<br>CCCGG	660

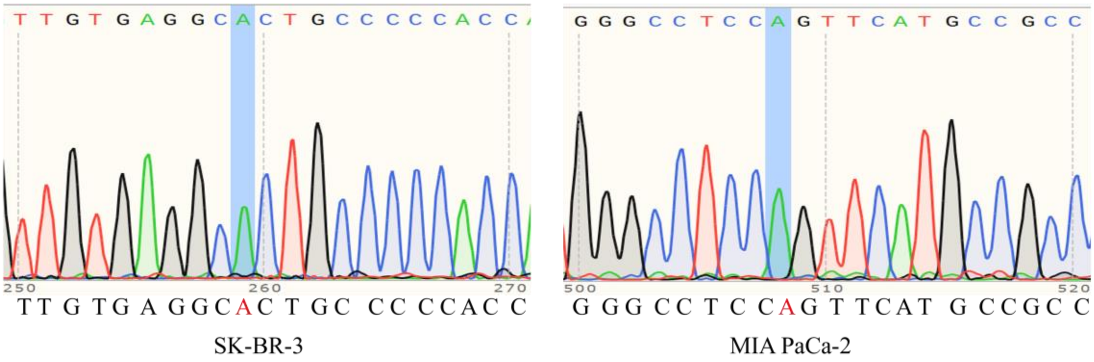

Figure S1. The sanger sequencing results of cells.

DPCR method optimization

The optimization of dPCR reaction conditions encompasses annealing temperature and primer/probe concentration. To optimize the annealing temperature, the primers and probes for TP53-R175H and TP53-R248W were respectively set to 500 nM and 250 nM, and four temperature gradients of 54 °C, 56 °C, 58 °C, and 60 °C were chosen. The results are presented in Figures S2 and S3. According to the two-dimensional scatter plot, both positive and negative droplets can be well distinguished at different annealing temperatures. 56 °C is determined as the optimal annealing temperature.

After the annealing temperature was determined, the concentrations of TP53-R175H and TP53-R248W primers and probes were optimized. The primer concentrations were set to 500 nM and 900 nM respectively, and the probe concentrations were set to 125 nM, 250 nM, and 500 nM respectively. The results are shown in Figures S4 and S5.

At the same primer concentration, as the probe concentration increased from 125 nM to 500 nM, the fluorescence intensity and separation degree of both positive and negative droplets increased, but the rainfall situation of positive droplets also became more severe. When the probe maintained a certain concentration and the primer concentration increased from 500 nM to 900 nM, there was no significant change in the fluorescence intensity of the positive and negative droplets. Therefore, the primer concentration for TP53-R175H and TP53-R248W was determined to be 500 nM, and the probe concentration was determined to be 250 nM.

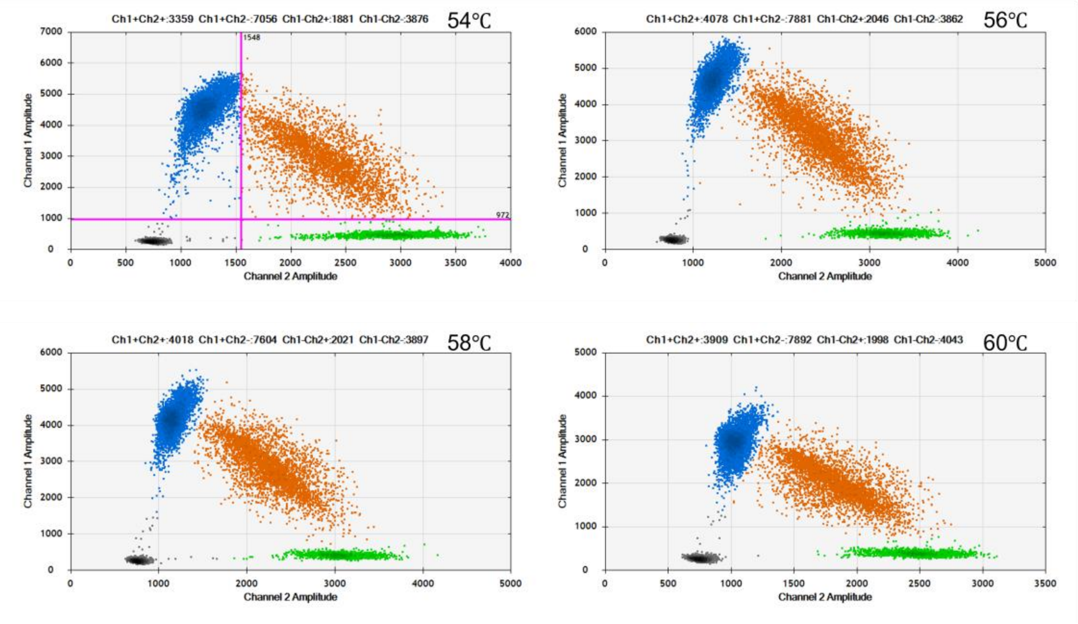

Figure S2. TP53-R175H annealing temperature optimization.

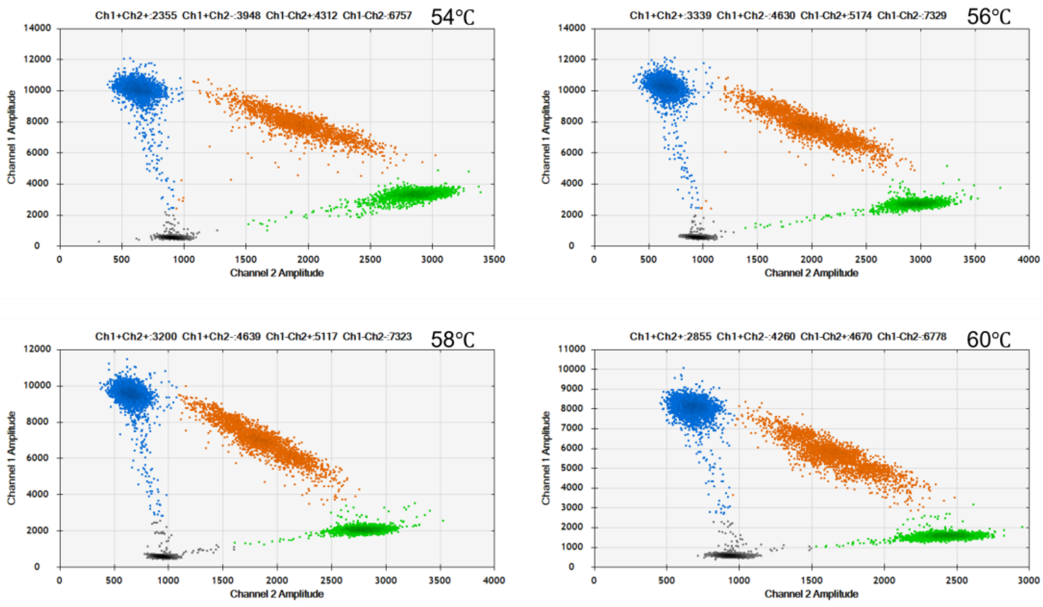

Figure S3. TP53-R248W annealing temperature optimization.

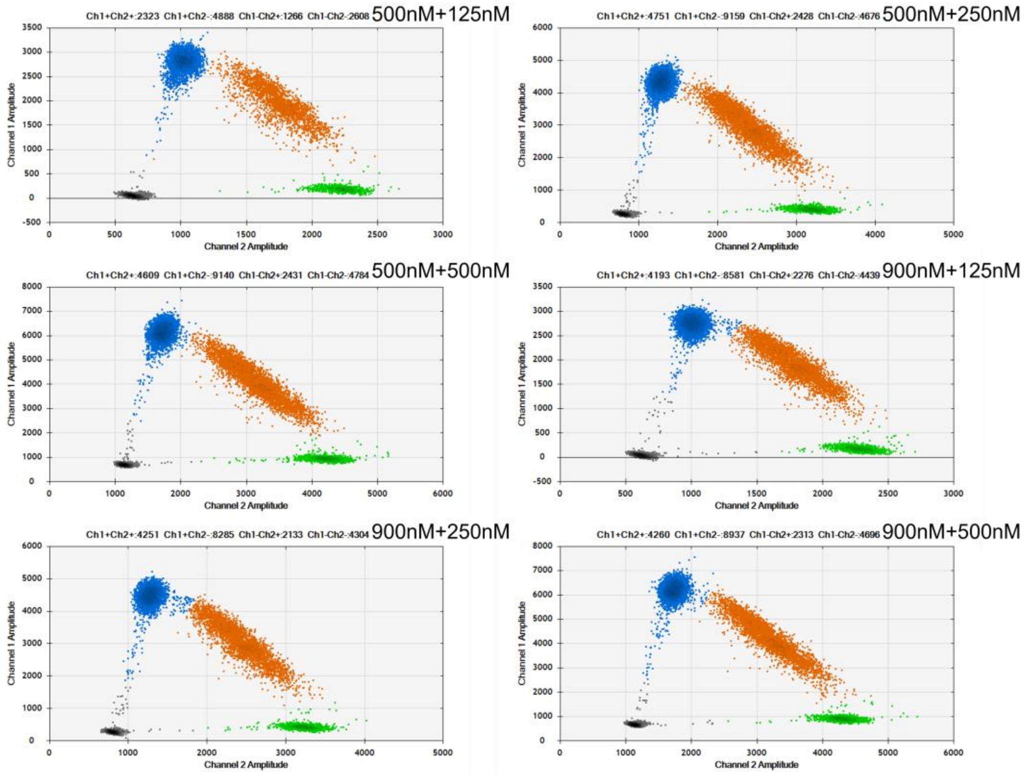

Figure S4. Optimization of primer and probe Concentrations for TP53-R175H.

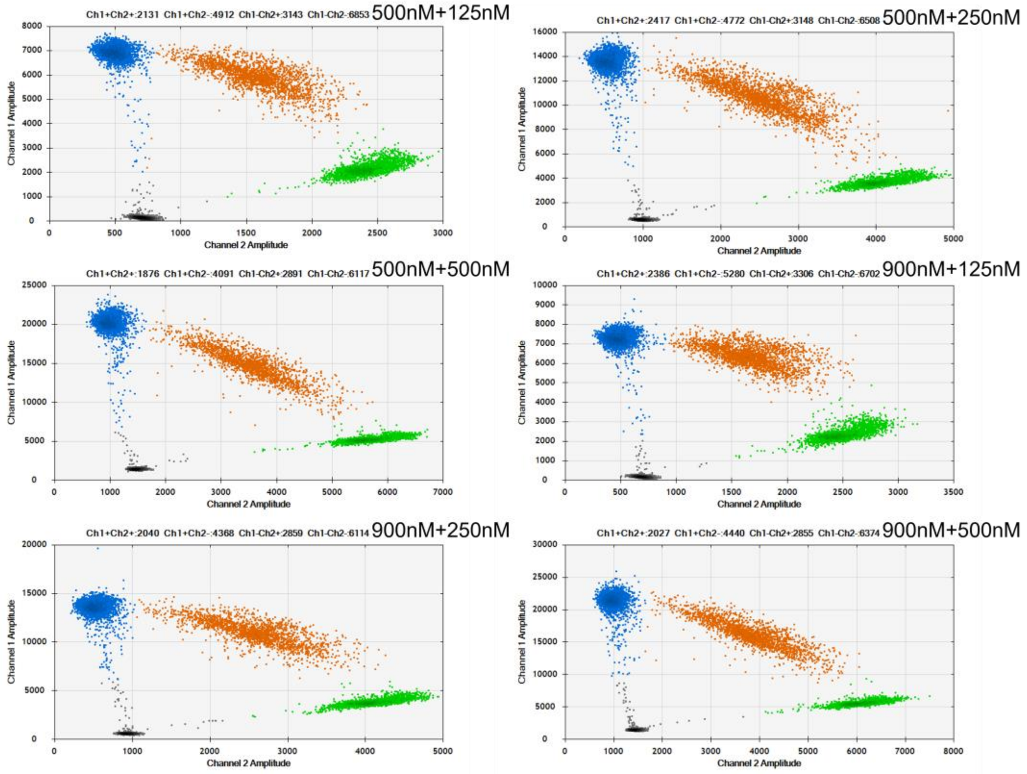

Figure S5. Optimization of primer and probe Concentrations for TP53-R248W.

Table S3. Raw dPCR data for the LOB of the TP53-R175H

Rank	Copy number concentration (cp/μL)		VAF	Rank	Copy number concentration (cp/μL)		VAF
	MU	WT			MU	WT	
**1**	0.11	780	0.0001	**31**	0.46	810	0.0006
**2**	0.11	764	0.0001	**32**	0.46	805	0.0006
**3**	0.17	767	0.0002	**33**	0.46	798	0.0006
**4**	0.17	764	0.0002	**34**	0.45	769	0.0006
**5**	0.19	784	0.0002	**35**	0.48	816	0.0006
**6**	0.22	780	0.0003	**36**	0.48	799	0.0006
**7**	0.23	773	0.0003	**37**	0.51	820	0.0006
**8**	0.23	767	0.0003	**38**	0.52	790	0.0007
**9**	0.24	761	0.0003	**39**	0.54	817	0.0007
**10**	0.26	780	0.0003	**40**	0.52	765	0.0007
**11**	0.26	778	0.0003	**41**	0.55	800	0.0007
**12**	0.28	796	0.0004	**42**	0.57	820	0.0007
**13**	0.29	808	0.0004	**43**	0.55	768	0.0007
**14**	0.29	800	0.0004	**44**	0.59	789	0.0007
**15**	0.3	806	0.0004	**45**	0.6	789	0.0008
**16**	0.29	777	0.0004	**46**	0.62	780	0.0008
**17**	0.3	792	0.0004	**47**	0.66	797	0.0008
**18**	0.3	771	0.0004	**48**	0.66	794	0.0008
**19**	0.31	767	0.0004	**49**	0.66	792	0.0008
**20**	0.33	803	0.0004	**50**	0.67	797	0.0008
**21**	0.33	780	0.0004	**51**	0.69	806	0.0009
**22**	0.35	793	0.0004	**52**	0.69	798	0.0009
**23**	0.35	773	0.0005	**53**	0.66	755	0.0009
**24**	0.36	783	0.0005	**54**	0.7	774	0.0009
**25**	0.35	760	0.0005	**55**	0.73	780	0.0009
**26**	0.36	775	0.0005	**56**	0.79	789	0.0010
**27**	0.37	781	0.0005	**57**	0.79	788	0.0010
**28**	0.39	788	0.0005	**58**	0.86	816	0.0011
**29**	0.41	814	0.0005	**59**	1.1	814	0.0013
**30**	0.39	755	0.0005	**60**	1.1	777	0.0014

Table S4. Raw dPCR data for the LOB of the TP53-R248W.

Rank	Copy number concentration (cp/μL)		VAF	Rank	Copy number concentration (cp/μL)		VAF
	MU	WT			MU	WT	
**1**	0	807	0.0000	**31**	0.18	754	0.0002
**2**	0	663	0.0000	**32**	0.19	793	0.0002
**3**	0	762	0.0000	**33**	0.19	763	0.0002

**4**	0	747	0.0000	**34**	0.2	767	0.0003
**5**	0	737	0.0000	**35**	0.2	733	0.0003
**6**	0.06	789	0.0001	**36**	0.22	766	0.0003
**7**	0.06	775	0.0001	**37**	0.23	774	0.0003
**8**	0.06	756	0.0001	**38**	0.24	806	0.0003
**9**	0.06	745	0.0001	**39**	0.24	798	0.0003
**10**	0.07	761	0.0001	**40**	0.23	760	0.0003
**11**	0.12	788	0.0002	**41**	0.24	792	0.0003
**12**	0.12	774	0.0002	**42**	0.23	746	0.0003
**13**	0.12	772	0.0002	**43**	0.23	745	0.0003
**14**	0.12	769	0.0002	**44**	0.24	775	0.0003
**15**	0.12	746	0.0002	**45**	0.23	738	0.0003
**16**	0.12	745	0.0002	**46**	0.24	757	0.0003
**17**	0.12	743	0.0002	**47**	0.24	756	0.0003
**18**	0.12	742	0.0002	**48**	0.25	776	0.0003
**19**	0.12	736	0.0002	**49**	0.26	805	0.0003
**20**	0.12	734	0.0002	**50**	0.26	783	0.0003
**21**	0.13	760	0.0002	**51**	0.29	783	0.0004
**22**	0.14	802	0.0002	**52**	0.31	779	0.0004
**23**	0.17	803	0.0002	**53**	0.31	746	0.0004
**24**	0.17	764	0.0002	**54**	0.32	763	0.0004
**25**	0.18	800	0.0002	**55**	0.35	825	0.0004
**26**	0.17	755	0.0002	**56**	0.35	770	0.0005
**27**	0.17	752	0.0002	**57**	0.43	814	0.0005
**28**	0.18	782	0.0002	**58**	0.42	795	0.0005
**29**	0.17	735	0.0002	**59**	0.45	750	0.0006
**30**	0.18	765	0.0002	**60**	0.56	781	0.0007

Table S5. Sensitivity analysis of the dPCR assays.

TP53-R175H			TP53-R248W		
Measure VAFs/%	Positive (N)	Total (N)	Measure VAFs/%	Positive (N)	Total (N)
0.077	1	12	0.020	0	12
0.082	2	12	0.031	1	12
0.116	8	12	0.065	10	12
0.128	10	12	0.100	11	12
0.333	12	12	0.204	12	12
